# Comparison of Biofilm Formation between Methicillin-Resistant and Methicillin-Susceptible Isolates of *Staphylococcus aureus*

**DOI:** 10.7508/ibj.2016.03.007

**Published:** 2016-07

**Authors:** Abdolmajid Ghasemian, Shahin Najar Peerayeh, Bita Bakhshi, Mohsen Mirzaee

**Affiliations:** 1 Department of Bacteriology, Faculty of Medical Sciences, Tarbiat Modares University, Tehran, Iran;; 2 Department of Laboratory Sciences, Borujerd Branch, Islamic Azad University, Borujerd, Iran

**Keywords:** Biofilm, Methicillin-resistant *Staphylococcus aureus*, Methicillin-susceptible *Staphylococcus aureus*

## Abstract

**Background:**

The aim of this study was to compare the biofilm formation and the prevalence of biofilm-associated genes between the isolates of methicillin-resistant (MRSA) and methicillin-susceptible (MSSA) *Staphylococcus aureus*.

**Methods:**

In total, 209 *S. aureus* isolates were collected. The antibiotic susceptibility test was conducted using nine antibiotics according to the guidelines of Clinical and Laboratory Standards Institute. Phenotypic biofilm formation was performed with microtiter plate assay. The polymerase chain reaction was employed to detect *icaA, icaD, icaB, icaC*, *clfA, clfB*, *fnbA, fnbB*, *fib*, *cna,* *eno*, *ebps*, *bbp,* *mecA*, and SCC*mec *types as well as *agr *group genes with specific primers.

**Results:**

Sixty-four (30.62%) isolates were resistant to methicillin, and 54 (83%) MRSA harbored SCC*mec* III. Furthermore, 122 (58.3%) isolates belonged to *agr* group I. Twenty-six (36.1%) MRSA and 42 (28.9%) MSSA isolates were strong biofilm producers (no significant difference). The prevalence of *icaA*, *icaD*,* icaB*, and* icaC* genes in MSSA isolates was 71, 41, 76, and 72%, respectively. The frequency of *clfA, clfB*, *fnbA, fnbB*, *fib*, *cna*, *eno*, *ebps*, and *bbp* in MSSA was 100, 100, 56, 46, 74, 54, 78, 11, and 1%, respectively. However, in MRSA isolates, the frequency was 97, 97, 64, 51, 76, 56, 79, and 12% with no track of *bbp*, respectively.

**Conclusion:**

Statistical difference between MSSA and MRSA regarding biofilm formation and the frequency of all biofilm-encoding genes was not significant. The majority of the *S. aureus* isolates harbored *clfA*, *clfB*, *eno*, *fib*, *icaA*, and *icaD* genes.

## INTRODUCTION

*Staphylococcus aureus* is one of the most nosocomial pathogens. Methicillin-resistant *S. aureus* (MRSA) strains, which have been developed for four decades, resist a wide spectrum of antibiotics. Biofilm formation in *S. aureus *isolates occurs through a polysaccharide intercellular adhesion (PIA) and also through microbial surface components recognizing adhesive matrix molecules (MSCRAMMs)^[^^1^^-^^4^^]^. These structures mediate the *S*.* aureus* initial attachment to both host tissues and biomaterials^[^^5^^]^. Biofilm formation interferes with bacterial recognition and killing mechanisms of the innate immune system^[^^4^^]^. MSCRAMMs play a key role in initiation of endovascular, bone and joint and prosthetic device infections^[^^6^^]^. Various *S.* *aureus* strains may not have a similar profile in the prevalent constellations of MSCRAMMs and also can make the individuals predispose to certain kinds of infections through binding to molecules such as collagen, fibronectin and fibrinogen^[^^7^^,^^8^^]^. *S. aureus* can express up to 20 different adhesive MSCRAMMs that are covalently anchored by sortase to peptidoglycan via the C-terminal LPXTG motif^[^^6^^]^. These adhesion proteins include the ClfA and ClfB (clumping factors A and B), FnbA and FnbB (fibronectin-binding proteins A and B), Fib (fibrinogen-binding protein), Cna (collagen-binding protein), Eno (laminin-binding protein), Ebp (elastin-binding protein), and Bbp (bone sialoprotein-binding protein). The initial site of attachment is in the moist squamous epithelium of the anterior nares of the host^[^^9^^]^. ClfA is the major *staphylococcal * fibrinogen binding-protein and is responsible for the observed clumping of *S. aureus* in blood plasma and culminating in arthritis and endocarditis^[^^4^^]^. ClfA also binds to platelet άIIbβ3 integrin^[^^7^^]^. On the other hand, ClfB binds to human cytokeratin 10 and to fibrinogen, as a bi-functional protein and thus mediates the nasal colonization and acts as a key virulence factor, which leads to metastatic infection and/or development of sepsis^[^^7^^]^. Both ClfA and ClfB interact with and inhibit complement C3. FnbA and FnbB are also involved in bacterial invasion of the endothelial cells *in vivo* and *in vitro* through RGD motif and then mediate the induction of cell signaling and reorganization of the actin cytoskeleton^[^^10^^]^. The FnBPA adhesions interact with receptors on endothelial cells and result in cardiovascular disease and cardiac device infections via platelet activation, a key step in thrombus formation and attachment to implanted prosthetic materials, respectively^[^^7^^]^. The *cna *gene encodes Cna protein and mediates the adhesion of *S. aureus* to collagenous tissues and cartilage^[^^11^^]^. It has been reported that the acquisition of antibiotic resistance does not change the *capacity* of *biofilm* formation in MRSA strains^[^^2^^]^. The aim of this study was to compare the prevalence of biofilm-related genes and their ability in biofilm formation between MRSA and MSSA isolates.

## MATERIALS AND METHODS

**Bacterial isolates**

**Fig. 1:**
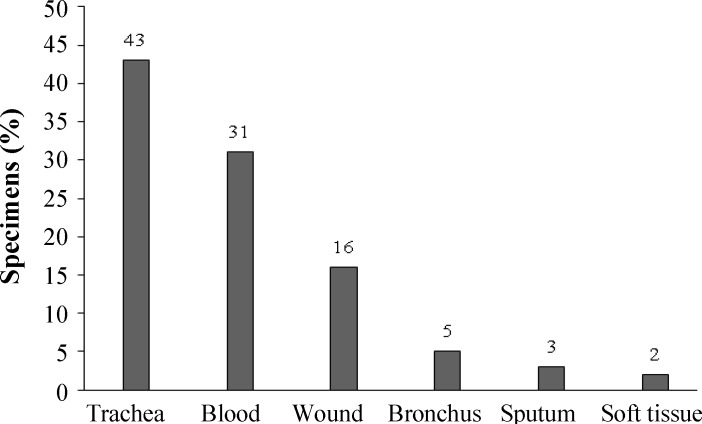
Different sampling sites for collection of the isolates

A total of 209 *S. aureus* clinical isolates were collected from patients in different hospitals from July 2012 to January 2013. Figure 1 shows the major sites of infection, including trachea, blood, wound, bronchus, sputum, and soft tissue. The isolates were identified using biochemical tests, such as mannitol fermentation on mannitol salt agar (Merck, Germany) medium, slide and tube coagulase tests, DNase production and colony morphology on blood agar medium. 

**Antibiotic susceptibility test**

Antimicrobial susceptibility test (AST) was performed according to the guidelines of Clinical and Laboratory Standards Institute^[^^12^^]^. The *S. aureus* strain of ATCC25923 was prepared to control the quality of the antibiotic susceptibility test. Different disks were used in AST, including oxacillin (1 µg), erythromycin (15 µg), clindamycin (2 µg), vancomycin (2 µg), linezolid (30 µg), tetracycline (30 µg), trimethoprim-sulfamethoxazole (25 µg), gentamicin (10 µg), amoxicillin (10 µg), and ciprofloxacin (5 µg) (all purchased from MAST, UK).

**Microtiter tissue plate (MTP) method**

The MTP method was conducted as previously described^[10]^. In brief, the wells of a microtiter plate were inoculated with 180 μl trypticase soy broth supplemented with 1% glucose. Each bacterial culture (20 µl) with a turbidity equivalent to an 0.5 McFarland standard was added to each well of polystyrene, 96-well, sterile, flat-bottomed tissue culture plates. After 24-h incubation at 35ºC, the wells were decanted and washed three times with sterile saline phosphate buffer. Next, methanol (for 20 min), and safranin 0.1% (for 15 min) were added to the wells. The stained wells were washed and left to ambient temperature to be dried. The safranin dye bound to the adherent cells was dissolved in 1 mL 95% ethanol per well, and the optical densities (ODs) of the plates were observed at 490 nm (A490) by using a microtiter-plate reader. Each assay was performed in triplicate. As a negative control, trypticase soy broth medium was used to determine background OD. OD cut-off was then determined as an average OD of negative control + 3× standard deviation (SD) of negative control. The OD cut-off value was separately calculated for each microtiter plate. Biofilm formation by isolates was calculated and categorized according to the absorbance of the safranin-stained attached cells (Table 1)^[^^10^^]^.

**Table 1:** Classification of biofilm formation abilities by MTP assay^[^^10^^]^

**Cut-off value calculation**	**Biofilm formation abilities**
OD>4×ODc	Strong
2×ODc<OD≤4×ODc	Moderate
ODc<OD≤2×ODc	Weak
OD≤ODc	Negative

**Fig. 2:**
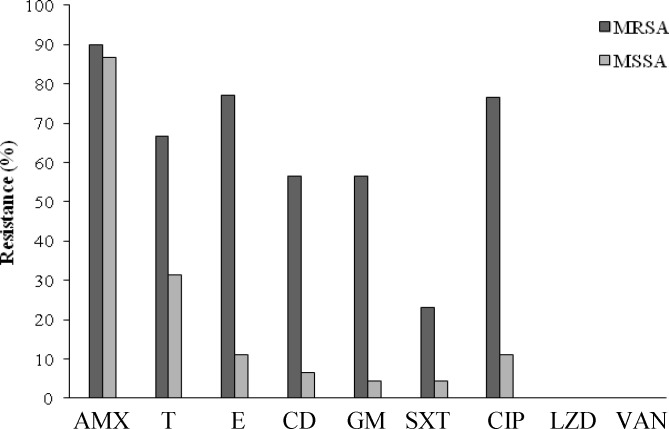
The antibiotic resistance pattern of MRSA and MSSA isolates. AMX, amoxicillin; T, tetracycline; E, erythromycin; CD, clindamycin, GM, gentamicin; SXT, trimethoprim-sulfamethoxazole; CIP, ciprofloxacin; LZD, linezolid; VAN, vancomycin

**Extraction of genomic DNA**

Five colonies of each bacterial isolate were suspended in 200 µl Tris-EDTA (TE) buffer and then 20 µl lysostaphin (2 µg/ml, Sigma, UK) was added. The genomic DNA of the isolates was purified according to the method described before^[^^13^^]^.

**Polymerase chain reaction (PCR)**

Simplex and multiplex PCRs were used to amplify *mecA* gene in MRSA, SCC*mec* types and *agr* groups as well as the genes encoding PIA and adhesive surface proteins, including *icaA*, *icaB*, *icaC*, *icaD,* *clfA, clfB*, *fnbA, fnbB*, *fib*, *eno*, *cna*, *ebps*, and *bbp*. Specific primers for these genes and also the thermal profiles of PCR for the studied genes have been depicted in our previous studies^[^^14^^,^^15^^]^.

**Data analysis**

Pearson’s chi-square was used to in statistical analysis. A *P* value less than 0.05 was considered statistically significant. 

## RESULTS

**Antibiotic susceptibility testing**

All the isolates were susceptible to vancomycin and linezolid. The antibiotic susceptibility pattern of the isolates for amoxicillin, erythromycin, tetracycline, gentamicin, clindamycin, ciprofloxacin, and trimethoprim-sulfamethoxazole was as follows: 90% (n=189), 29% (n=61), 25% (n=52), 19% (n=40), 19% (n=40), 15.3% (n=32), 11% (n=23), respectively. 

**Detection of methicillin-resistant** ***Staphylococcus aureus*** **strains**

The phenotypic test indicated that 64 (30.62%) isolates were resistant to methicillin. The *mecA* gene was also detected in these isolates with specific primers. The MRSA isolates were significantly more resistant than MSSA isolates to the majority of the antibiotics (*P*≤0.05, Table 2), except for vancomycin and linezolid (Fig. 2). 

**Phenotypic biofilm production**

The MTP assay demonstrated that 14 (21.8%) MRSA and 42 (28.9%) MSSA isolates were strong biofilm producers (no significant difference). Furthermore, approximately 50% of the total isolates showed a moderate level of biofilm formation. 

**SCC** ***mec*** **types**

The majority of the MRSA isolates (n=54, 84%) harbored SCC*mec* III. However, 30 isolates with SCC*mec* type III were only susceptible to vancomycin and linezolid. The SCC*mec* types V and I were detected in 9% (n=6) and 6% (n=4) of the isolates, respectively.

***agr*** **genes**

In total, 122 (58.3%) isolates belonged to *agr* group I, followed by *agr* group II (n=46, 22%), *agr* group IV (n=27, 13%) and *agr* group III (n=9, 4%). There was no relationship between *agr* specific groups and clinical signs (*P*=0.21).

**Frequency of** ***icaA, icaD, icaB*** **and** ***icaC*** **genes**

**Fig. 3:**
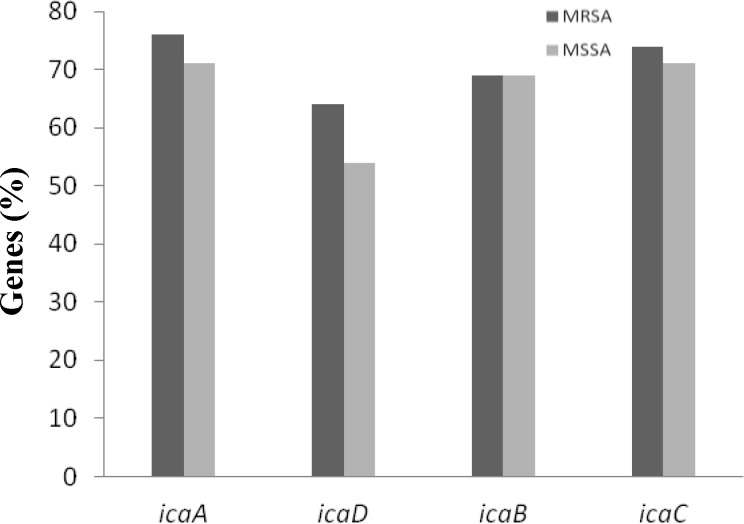
The frequency of the *icaA*,* icaD*,* icaB*, and* icaC* genes between MRSA and MSSA

The *ica *genes were identified by PCR method. The size of PCR product for *icaA*,* icaD*,* icaB*, and* icaC* genes were 188, 198, 900, and 1100 bp, respectively. The frequency of the *icaA*,* icaD*,* icaB*, and* icaC* genes in MSSA isolates was 71%, 54%, 69%, and 71%, respectively. In the MRSA isolates, the frequency of these genes was 76%, 69%, 64%, and 74%, respectively. There was no significant difference between MRSA and MSSA regarding the presence of these genes. Also, there was no relation between *icaA, icaD*, *icaB*, *icaC* genes and *agr* groups or MRSA. The difference in the frequency of *icaA, icaD, icaB*, and *icaC* genes between MRSA and MSSA is shown in Figure 3. 

**Table 2:** Comparison between MSSA and MRSA isolates regarding antibiotic resistance percentage and the prevalence of *agr *group I

**Isolates**	**AMX**	**CD**	**E**	**T**	**SXT**	**GM**	**CIP**	***agr *** **I**
MSSA	86.66	6.66	11.11	31.11	4.44	4.44	11.11	56%
MRSA	90.00	56.66	77.00	66.66	23.23	56.66	76.66	58%
*P*	0.57	0.001	0.001	0.03	0.02	0.001	0.001	0.52

**Prevalence of genes encoding microbial surface components recognizing adhesive matrix molecules**

**Fig. 4:**
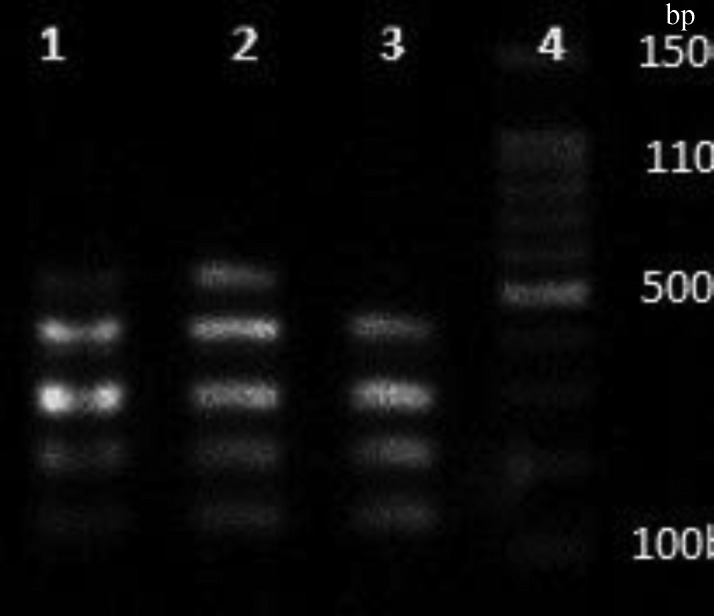
The PCR products of *clfA*, *clf*B, *fnbA*, *fnbB,* and *fib* genes. Lanes 1 and 2, *clfA* (288 bp), *clfB* (204 bp), *fnbA* (128 bp), *fnbB* (524 bp), and *fib* (405 bp) genes; Lane 3, all the genes without the *fnbB* gene; Lane 4, marker 100 bp (Fermentas, USA).

**Fig. 5.**
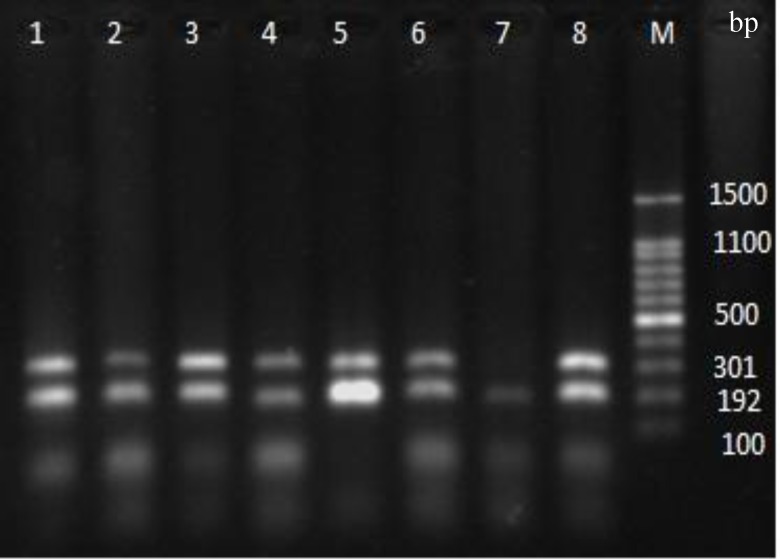
The multiplex of *eno*, *cna*, *ebps*, and *bbp* genes. Lanes 1-6, and 8, *eno* and *cna* genes with 301 and 192 bp, respectively; Lane 7, *cna* gene (the *ebps* and *bbp* genes not shown). M: marker 100 bp (Fermentas, USA).

**Fig. 6:**
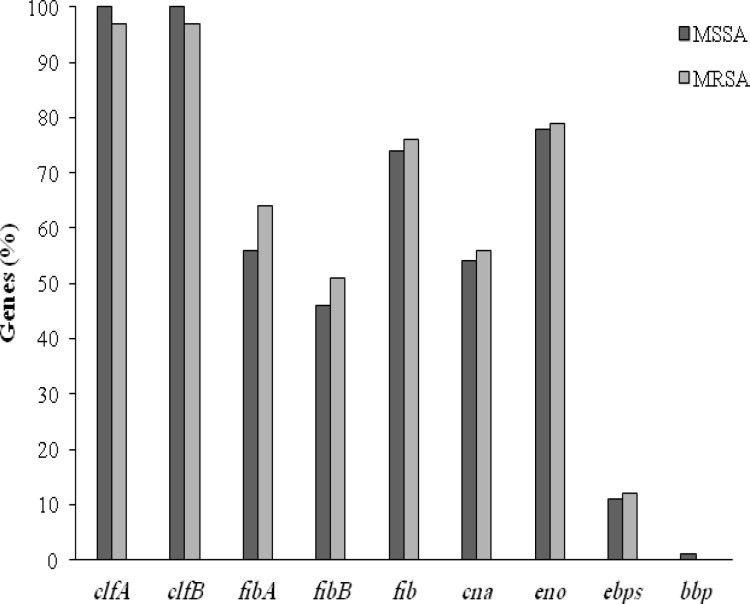
The frequency of the genes encoding MSCRAMMs between MRSA and MSSA isolates.

Biofilm-related genes were amplified by two multiplex PCR panels (Figs. 4 and 5). The frequency of *clfA, clfB*, *fnbA, fnbB*, *fib*, *cna*, *eno*, *ebps*, and *bbp* in MRSA isolates was 97%, 97%, 64%, 51%, 76%, 56%, 79%, and 12% with no track of *bbp*, respectively. However, in MRSA isolates, the frequency was 100%, 100%, 56%, 46%, 74%, 54%, 78%, 11%, and 1%, respectively (Fig. 6). The statistical difference between MSSA and MRSA regarding the frequency of all the biofilm-encoding genes was not significant (Table 3). There was no relationship between these genes and *agr* groups as depicted in Table 4.

## DISCUSSION

All of the isolates in the present study were susceptible to vancomycin and linezolid and likewise, the majority was susceptible to trimethoprim-sulfamethoxazole. Generally, vancomycin and linezolid are completely effective in MRSA treatment; however, reduced susceptibility to both antibiotics have been reported in some studies^[2,16]^. Vancomycin and other glycopeptides have remained the last resorts for eradication of *S. aureus* infections. 

The results from the current study indicated that the antibiotic susceptibility pattern of the isolated pathology originated from different infected areas (trachea, blood, wound etc.) was not significantly meaningful. Also, 64 isolates were MRSA, and the majority of MRSA harbored the SCC*mec* type III. Previous reports have also indicated that the SCC*mec* type III is the predominant type in Iran^[^^2^^,^^16^^,^^17^^]^. Based on our findings, the majority of the isolates (both MRSA and MSSA) belonged to *agr* I (58.3%), followed by the *agr II*, *agr* IV, and the *agr* III. Previous studies have also depicted that the *agr *I is the predominant type in Iran^[^^15^^,^^18^^]^. The relationship between *agr *I and several characteristics, such as the AST pattern, the prevalence of biofilm-related genes, and biofilm formation exhibited that the isolates belonged to *agr *I had higher antibiotic resistance compared to those with other *agr* specific groups. In the phenotypic biofilm formation, the MSSA and MRSA isolates produced biofilm, and there was no significant difference (*P*<0.05).

The present study indicated that ClfA and ClfB were present in more than 95% of the isolates and constituted the bound coagulase of *S. aureus*. Similar to the current study, previous surveys have determined a high prevalence of *icaA and icaD* genes with a relationship to phenotypic biofilm formation^[^^19^^-^^21^^]^. For instance, Nasr *et al.*^[^^21^^] ^detected the *icaA and icaD* genes in (34%) of catheter and blood isolates that were capable of biofilm formation. This study demonstrated that there was a relationship between the biofilm formation and the presence of these genes. The difference between MRSA and MSSA was not significant regarding the presence of these genes. This result emphasizes that the SCC*mec* genes are separate from and independent of *ica* locus. A comparative analysis between these isolates in the present study demonstrated that there is no significant difference between blood, wound and trachea isolates regarding the presence of biofilm-associated genes. The frequency of the *clfA, clfB*, *fnbA, fnbB*, *cna*, *eno*, *fib*, *ebps*, and *bbp* genes between MRSA and MSSA isolates was not significantly different (Fig. 6), which is similar to the Atshan *et al.*^[^^5^^]^ result. George *et al.*^[^^20^^] ^have found that those isolates lacking *clfA* has lower have found that those isolates lacking *clfA* has lower ability in binding to fibrinogen. Moreover, specific clonal complexes of *S. aureus* may contain different prevalence profiles of MSCRAMMs^[^^22^^]^. Biofilm formation is influenced by a variety of conditions and regulatory factors. In this study, isolates collected from different clinical samples had no significant difference regarding the presence of these genes. However, the differences in the results of various studies may be influenced by the epidemiological factors, genetic background of isolates, origins of transmission and other factors. Pozzi *et al.*^[^^23^^]^ reported that biofilm formation in MSSA mainly occurs via PIA synthesis while in MRSA, it is more related to the *fnbB *adhesion. 

**Table 3:** The comparison of biofilm-related genes percentage between MRSA and MSSA isolates

**Isolates**	***icaA***	***icaB***	***icaC***	***icaD***	***clfA***	***clfB***	***fnbA***	***fnbB***	***cna***	***eno***	***fib***	***ebps***	***bbp***
MSSA	71.00	54.00	69.00	71.00	100.00	100.00	56.00	46.00	54.00	78.00	74.00	11.00	1.00
MRSA	76.00	64.00	69.00	74.00	97.40	97.40	64.00	51.00	56.00	79.00	76.00	12.00	0.00
*P value*	0.37	0.26	0.59	0.42	0.57	0.57	0.32	0.33	0.56	0.34	0.36	0.43	0.53

In this study, the prevalence of *fnbA and fnbB* genes was higher in MRSA. Furthermore, this study demonstrated that the biofilm production may be independent of the *agr* specific groups. To our knowledge, there are no previous studies to detect the relationship between *agr* function and the ability in *ica* biofilm formation in clinical isolates of *S. aureus*.

In conclusion, this study reports that there is no relation between antibiotic resistance and biofilm formation in clinical isolates of *S. aureus* and also there is no correlation in the distribution of MSCRAMMs and biofilm genes with biofilm formation *in vitro*.

**Table 4:** The presence of biofilm-related genes in case of each *agr* specific group

**Group**	***icaA***	***icaB***	***icaC***	***icaD***	***clfA***	***clfB***	***fnbA***	***fnbB***	***cna***	***eno***	***fib***	***ebps***	***bbp***
*agrI*	59	45	61	61	98	98	65	45	57	90	70	12	0.02
*agrII*	54	49	56	54	100	100	74	44	62	92	69	13	0
*agrIII*	79	64	71	70	100	100	76	43	56	76	66	7	0
*agrIV*	67	60	67	69	100	100	67	33	67	80	70	7	0
